# Factors Influencing Adhesive Bonding Efficiency in ETICS Application

**DOI:** 10.3390/ma18174043

**Published:** 2025-08-29

**Authors:** Paweł Gaciek, Mariusz Gaczek, Paweł Krause

**Affiliations:** 1 Faculty of Civil Engineering Silesian University of Technology, 44-100 Gliwice, Poland; pawel.krause@polsl.pl; 2 BOLIX S.A., 34-300 Żywiec, Poland; 3 Faculty of Civil and Transport Engineering, Poznan University of Technology, 60-965 Poznan, Poland; mariusz.gaczek@put.poznan.pl

**Keywords:** ETICS, adhesive layer thickness, cement-based adhesive mortar, ribbon-and-dab method, contact surface area

## Abstract

In this study, physical factors influencing the efficiency of adhesive bonding in External Thermal Insulation Composite Systems (ETICS) using the ribbon-and-dab bonding method were analyzed. Tests were carried out to show the distribution of pressure transmitted through thermal insulation to adhesive mortar and substrate during bonding, and to demonstrate the relationship between pressure, adhesive layer thickness, and bond strength of mortar to concrete substrate. The analysis was also based on in situ observations, laboratory experiments, and numerical modeling, with particular attention paid to contact pressure and adhesive strength depending on cement-based mortar layer thickness. Example pull-off tests (CAST, DAST) performed on dabs showed that increasing thickness from 10 mm to 20 mm caused a decrease in bond strength in the central area by about 86% for tested adhesive mortars and substrate—values dropped from 1.8 MPa to below 0.25 MPa, while edge zones often showed no adhesion. Pressure-mapping tests (PMAST) revealed distinct pressure zones within dabs and perimeter ribbons. The analysis showed that average normalized pressures in adhesive dabs reached about 52% of the maximum value, while the [0.9; 1.0] pressure interval covered about 12% of the contact area. Based on empirical data, a decay function was developed to build a model of radial pressure attenuation. Monte Carlo simulations defined ranges of random model parameters and variability of average pressures in a 10 mm adhesive dab. The model allowed inclusion of a peripheral zero-pressure ring and enabled simulation for a 20 mm layer, confirming that increased thickness led to reduced contact pressure and explained the decrease in bonding performance.

## 1. Introduction

High thermal insulation of external building walls has long been a key priority in shaping energy-efficient and environmentally sustainable residential construction in Europe [1]. One of the most widely adopted solutions is the External Thermal Insulation Composite System (ETICS). This system provides a continuous and effective thermal insulation layer while also contributing to the decorative architectural appearance of the building [2]. ETICS enables effective protection against various environmental factors such as surface contamination, microbiological growth, and elevated surface temperatures on surfaces with low solar reflectance, which can be mitigated through the use of cool pigments [3]. These systems are applicable to both new and existing buildings, regardless of the wall construction materials used [4]. The combination of these advantages—including improved energy efficiency, reduced greenhouse gas emissions, and alignment with the EU’s full decarbonization goals [5]—has contributed to the sustained popularity of ETICS in residential construction across Europe.

In recent years, additional factors have expanded the possibilities for architectural design using ETICS on external walls, while simultaneously introducing new challenges regarding the long-term durability of such systems. This is largely due to the increased architectural complexity of ETICS finishes, which significantly raises the self-weight of the insulation system through the use of stone or ceramic cladding, various types of panels, or decorative elements [6,7,8]. Similarly, varying surface loads across different ETICS zones—resulting from decorative features with differing mass, thickness, or thermal deformability—can create additional complications [9]. Another key issue involves thermal stresses on external render surfaces caused by very dark colors, which strongly absorb solar radiation [10], or at junctions with lighter-colored renders that have high light reflectance values (HBW) [11]. Further challenges arise from the increasing diversity of insulation materials and their combinations [12], as well as variations in mechanical fixings [13], and their impact on localized moisture transfer in disrupted ETICS zones [14]. Therefore, it is no longer accurate to assume that a previously validated and well-studied technology remains universally applicable in its original form. The vast scale of ETICS implementation across Europe, along with its dynamic evolution, highlights the need to reassess established solutions under these changing conditions.

This need for reassessment is consistent with the current state of research, where most studies on ETICS have concentrated on aspects other than adhesive bonding.

The majority of publications address hygrothermal performance and moisture safety, fire resistance of insulation systems, durability of rendering layers under weathering, or acoustic performance of building envelopes. In contrast, only a few works have dealt directly with adhesive bonding efficiency and the mechanics of bonded areas. In [15], the realistic load-bearing behavior of ETICS under wind suction was investigated, demonstrating that systems with partial bonding failed much earlier than expected, whereas fully bonded systems exhibited significantly higher resistance. In [16], the adhesion of ETICS to timber-based panels was studied, showing that bond strength strongly depends on substrate characteristics, with failures sometimes occurring within the substrate itself. More recently, [17] proposed a computer-vision-based quality inspection model for EIFS/ETICS adhesives, enabling automatic assessment of ribbon width, dab size, and bonded area with high accuracy.

These contributions underline that adhesive bonding plays a decisive role in the mechanical reliability of ETICS, yet systematic investigations of local contact pressure distribution, adhesive layer thickness, and intra-dab variability remain scarce. This paper attempts to fill this gap by combining in situ observations, laboratory investigations, and numerical modeling to quantify the effect of adhesive layer thickness and application method on effective bonding efficiency. Particular emphasis is placed on contact pressure distribution and local bond strength, which have not been systematically investigated in previous studies.

As demonstrated by construction practice, ETICS applications are associated with a significant number of execution-related defects [8,18,19,20,21]. One of the most persistent issues—observed for both new and existing buildings—is the unevenness of the substrate (i.e., wall surfaces) [22]. Surface irregularities have a direct impact on the variability and, in most cases, excessive thickness of the adhesive layer used to bond the thermal insulation [23].

The issue of wall surface unevenness in multi-story buildings—including newly constructed buildings—is increasingly becoming a source of non-compliant practices. The cost estimation procedures for ETICS installation are usually standardized and rarely account for the proper correction of wall irregularities. Yet, such correction is both possible and necessary, for example, by utilizing leveling mortars or adjusting the thickness of the thermal insulation layer. However, when these measures are treated as additional work, they introduce extra costs that neither investors nor contractors are typically willing to bear. As a result, the most common practice in the field is to compensate for substrate unevenness by increasing the thickness of the adhesive layer [24].

Despite the extensive experience accumulated in Europe regarding both testing methodologies and practical implementation of ETICS, there is a lack of clear methods to define the maximum acceptable thickness of adhesive layers used to bond thermal insulation to the substrate. Such information is typically provided by adhesive manufacturers in technical datasheets or on packaging. Adhesive mortars available in the Polish market specify a variety of acceptable layer thicknesses, most commonly ranging from a few millimeters up to 10 mm. According to [22,24], adhesive layers can be used to compensate for substrate irregularities up to 10 mm in fully bonded systems, and up to 20 mm in systems that combine adhesive and mechanical fixings. Guidelines [25] state that the adhesive layer bonding thermal insulation to the substrate should not exceed 20 mm, provided that sufficient adhesion is achieved to both the substrate and the insulation material, and that the effective bonded surface area is no less than 40%. Although such conditions are specified, the guideline ultimately refers the user to the adhesive layer thickness recommended by the manufacturer of the specific product.

Some manufacturers do not specify the thickness of the adhesive layer at all, providing only the consumption rate per square meter of insulation and the minimum required coverage area on the board—typically at least 40%, particularly on the Polish market. Detailed calculations based on these values indicate that, in most cases, the adhesive layer can be only a few millimeters thick. Explicit recommendations regarding adhesive thickness are rarely found in Technical Assessments issued by national Technical Assessment Bodies for ETICS systems. This stands in contrast to other layers of the system—such as the thickness of the reinforced base coat, finishing render, or insulation—which are typically defined in detail. Furthermore, the recommended substrates for specific adhesives are often not clearly specified by type. This may suggest either that substrate characteristics are considered insignificant for adhesion, or that the current testing method defined in EAD 040083-00-0404 [26] is deemed sufficient for all substrates and adhesive layer thicknesses. However, practical experience shows that this is not the case.

For many years, various methods have been used to modify the surface properties of substrates, such as cleaning, dust removal, washing, and priming. These treatments reduce absorbency, enhance surface cohesion or adhesion, and improve the bonding performance of adhesives. Such an improvement of properties is most often recommended for those substrates that require it. In ETICS applications, it is also recommended to assess the substrate and adhesive bond strength using appropriate tests, including pull-off testing. However, national guidelines lack precise instructions on how to perform such tests in situ, for example, on vertical surfaces. In Poland, the so-called polystyrene cube gluing test is also practiced as an approximate method for testing the tensile load-bearing capacity of the substrate via the adhesion of the adhesive to both the substrate and polystyrene. The test uses EPS TR 100, which has a tensile strength perpendicular to its face of 100 kPa. The procedure is described in [25,27] and in earlier guidelines [28,29,30]. In this method, EPS cubes measuring 10 cm × 10 cm are bonded to selected areas of the assessed substrate (typically the surface of a wall) using an adhesive intended for thermal insulation. After several days, the blocks are manually pulled off perpendicular to the wall surface. The tensile capacity of the EPS serves as a measure of the substrate’s minimum tensile load-bearing capacity, mediated by the adhesive bond, which is, in a sense, enforced by the high application pressure. A cohesive failure within the EPS material is considered a positive result. It must be clearly stated that, due to the way the EPS blocks are bonded and pressed onto the surface, the test evaluates the load-bearing capacity of the substrate rather than the bond strength of the adhesive to the substrate—a distinction that is easy to confuse.

It is evident that the operational safety of thermal insulation systems depends to a large extent on their secure attachment. When adhesive-only fixing is used, the bond strength of the adhesive mortar to the substrate is crucial, as it constitutes the sole means of attachment. One of the methods of fixing ETICS is also mechanical fastening with additional adhesive. In this case, the concentration of loads (environmental actions) acting on ETICS relates to the effectiveness of the mechanical fastening, which is intended to transfer them. It is also worth emphasizing a formal aspect concerning mechanical fixings: EAD 330196-00-0604 [31] states that fixings are tested only for their performance under tensile loads—that is, mainly wind pressure, or more precisely, wind suction. In contrast, the self-weight of the insulation system is expected to be carried by the adhesive layer. This further highlights the importance of the adhesive’s role in ETICS attachment systems [32].

A key question, then, is what factors—apart from the previously mentioned surface modification methods—determine the quality of adhesion between the thermal insulation and the substrate?

The most common method of bonding thermal insulation to the wall in ETICS is the so-called ribbon-and-dab method [25]. This technique allows for minor adjustment to compensate for irregularities in the substrate surface during installation [25,27]. In this method, the adhesive is applied to the insulation board in two geometric forms. The first is a perimeter ribbon—a continuous bead of adhesive applied around the entire edge of the board on the contact side. The second involves adhesive dabs, placed symmetrically within the central area of the board. For boards measuring 50 cm × 100 cm, this usually means applying between 3 and 6 dabs. The minimum effective bonding area between the board and the substrate should be no less than 40% [25,27]—as per the Polish national guidelines. EAD 040083-00-0404 [26] indicates a minimum bonding area of 20%. Important features of the described method of bonding thermal insulation include the above-mentioned possibility of correcting certain wall irregularities and the possibility of rational distribution of the mortar on the board. For these reasons, it is used wherever ETICS is applied to masonry walls, cast-in-place concrete (reinforced concrete), prefabricated elements, mineral substrates, and similar types. This therefore applies to implementations throughout Europe, not only in Poland and the United Kingdom, where in situ investigations were carried out.

The most commonly and widely used adhesive for fixing thermal insulation is a polymer-modified cementitious mortar, which is economically and functionally justified. Cement adhesive is significantly cheaper than dispersion adhesive. It allows for the formation of thicker layers (joints) than dispersion and polyurethane adhesives. It provides good stabilization of the thermal insulation already during installation. Therefore, this type of mortar is the focus of the authors’ investigation into the bonding of thermal insulation to wall substrates.

Critical to understanding the issues analyzed in the manuscript were the actual methods used for bonding and, consequently, the technical condition of existing ETICS systems, as well as the qualitative assessment of the contact surfaces: adhesive–wall substrate and adhesive–insulation. Another important area of investigation was the bond strength of the adhesive to the substrate, depending on its geometric distribution and layer thickness. All tests were designed to show the distribution of the force transmitted through the thermal insulation to the adhesive mortar and the substrate during the actual bonding of the insulation. Another aim of the study was to determine the relationship between contact pressure, the distribution of its intensity, the thickness of the adhesive layer, and the bond strength of the adhesive mortar to the substrate. Complementing the whole was a numerical model that allowed determination of the variable pressure function within a single adhesive dab, which, as the tests showed, translated into a variable state of adhesion of the mortar to the substrate in its different parts. Among the known methods of assessing the technical condition of ETICS insulation—such as in situ testing—there were also original laboratory methods developed to determine bonding effects almost entirely comparable to those occurring in real ETICS applications.

## 2. Materials and Methods

### 2.1. In Situ Investigations

The subject of the investigation was an ETICS (External Thermal Insulation Composite System) applied to external walls, using thermal insulation made of expanded polystyrene (EPS) and mineral wool (MW), bonded to the substrate with a cement-based adhesive mortar. The study included buildings with varying levels of external wall thermal performance and different insulation thicknesses. In selected cases, mechanical fixings were also present in the system. The investigation primarily focused on traditional masonry wall structures built from small-format units, such as solid bricks, ceramic blocks, calcium silicate blocks, and autoclaved aerated concrete blocks. Additionally, in selected cases, the external walls were made of precast concrete panels. The ETICS finishing layer consisted of a thin-coat render (mineral, silicone, silicate, or acrylic), applied over the reinforced base coat.

**Figure 1:**
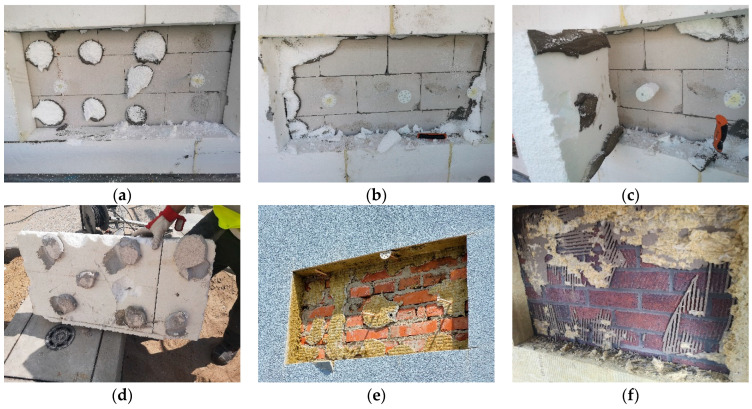
Examples of in situ tests—views after removal of thermal insulation boards from the wall: (**a**–**d**) expanded polystyrene (EPS); (**e**,**f**) mineral wool (MW).

Investigations were conducted on several dozen multi-family residential buildings, located mostly in various regions of Poland, and additionally at selected sites in the UK. The testing method involved cutting out fragments of the ETICS approximately 50 cm × 100 cm in size, which served as test samples. A macroscopic assessment was carried out to evaluate the pattern of adhesive distribution on the insulation board, the thickness of the adhesive joint, and the adhesive surface formed after bonding the insulation to the substrate. The sample dimensions were selected to ensure representative results across the full surface area of individual insulation boards. Based on this, a visual inspection was first performed to assess the application method of the adhesive (i.e., whether it followed the ribbon-and-dab pattern, a point-only application, or another method). The next step involved a macroscopic assessment of the separation pattern between the thermal insulation and the wall substrate. The geometric configuration of the adhesive bond between the substrate and the insulation material was analyzed, and the effective contact area was estimated. The effective bonding area was defined as the approximate projected area of the insulation that remained adhered to the hardened adhesive layer after removal. This allowed for the calculation of the effective percentage of contact between the adhesive and the insulation. Examples of selected failure patterns following mechanical removal are shown in Figure 1.

### 2.2. Laboratory Investigations

#### 2.2.1. Contact Surface Analysis Using the Original GCAT Method

**Figure 2:**
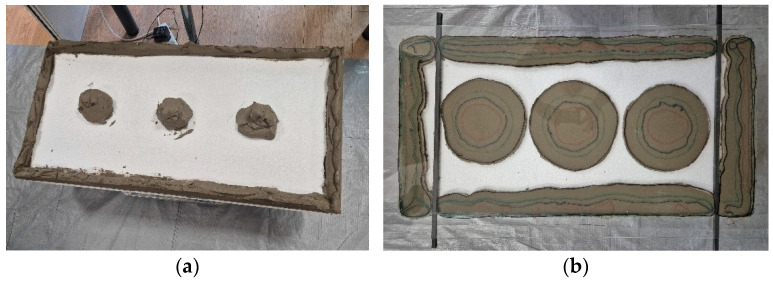
Glass Contact Area Test (GCAT): (**a**) EPS board with freshly applied adhesive; (**b**) glass pane placed on the adhesive-coated surface at the smallest spacer distance (10 mm), with colored outlines marking contact areas corresponding to greater adhesive thicknesses (30 mm, 20 mm, and 15 mm).

The contact surface between the adhesive and the substrate was evaluated using a proprietary test method called GCAT (Glass Contact Area Test), developed by Paweł Gaciek [33]. For this assessment, EPS TR 100 façade insulation boards (15 cm thick, 50 cm × 100 cm in size) were used. A cement-based adhesive mortar, being a component of the thermal insulation system for which a Technical Assessment was issued, was used for the test. Bulk density of the mortar: 1.44 g/cm^3^, water-to-mortar ratio: 0.20 L/kg. Approximate composition of the mortar: dry mixture of cement binder, polymers, fine-grained mineral fillers, and modifying additives. The adhesive mortar was applied manually to the board using the ribbon-and-dab method, in the form of a fresh mix weighing 6 kg. The insulation board with the applied adhesive was then placed horizontally, with the adhesive layer facing upward. Longitudinal closed-section profiles were placed on the insulation board to allow discrete adjustment of the adhesive layer thickness. The profiles were configured to provide specific thickness levels of 30, 20, 15, and 10 mm. Reducing the adhesive thickness was achieved by successively removing selected spacer profiles. After setting the spacer profiles to the maximum distance of 30 mm, a rigid glass pane—serving as a substitute substrate in this test—was placed parallel to the insulation board with the applied adhesive. A marker outline of the insulation board was drawn on the glass pane beforehand, and the pane was then positioned so that the outline matched the dimensions of the insulation board. Once pressed into place, the resulting adhesive contact area visible on the glass surface was traced with a colored marker. Next, the distance between the glass pane and the insulation board was reduced to 20 mm by removing one of the stacked spacer profiles. The board was pressed against the remaining spacers, and the resulting adhesive contact area was again outlined on the glass pane using a marker of a different color. The procedure was also repeated for adhesive layer thicknesses of 15 mm and 10 mm. In this way, using the same amount of adhesive, the contact areas between the adhesive mortar and the substitute substrate were determined for different adhesive thicknesses. All operations were carried out under laboratory conditions within a relatively short time frame to prevent the mortar from thickening during the test. The contact areas were estimated using the ZWCAD program [34]. An example of the GCAT procedure, including the resulting contact area markings on the glass pane, is shown in Figure 2.

#### 2.2.2. Testing Using the PMAST Method (Pressure Mapping Adhesive Spot Test)

To verify the mechanisms observed during the in situ investigations, a series of qualitative tests was designed to answer the following research question: How does the pressing force applied during thermal insulation bonding influence the adhesive, the substrate, and the insulation material, and what is the distribution of this force during application? The study was conducted using an original method, named PMAST (Pressure Mapping Adhesive Spot Test), developed by Paweł Gaciek [35].

The study employed a SENSOR MEDICA baropodometric platform, model FreeMED PROFESSIONAL (DMFM18050, DMS Group, Brignoles, France), equipped with a pressure-sensing mat measuring 1840 mm × 740 mm. The mat simulated a real wall substrate and enabled the generation of pressure maps. Inside the mat are square-shaped resistive sensors coated with 24-karat gold and conductive rubber, with an active sensing area of at least 1800 mm × 500 mm. The sampling frequency is at least 200 Hz for dynamic measurements and 150 Hz for static ones. The maximum load capacity is 1.5 MPa, and the operating temperature range is 0 °C to 55 °C.

**Figure 3:**
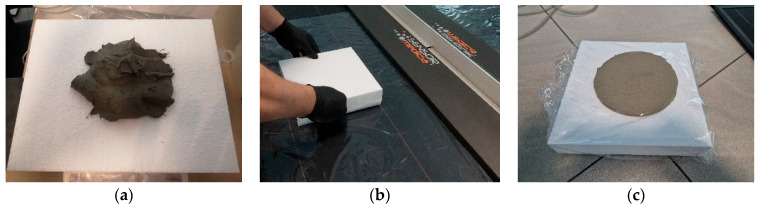
Pressure Mapping Adhesive Dab Test (PMAST)—tests on 25 cm × 25 cm samples: (**a**) test specimen before pressing—EPS board with a measured amount of fresh adhesive; (**b**) manual pressing of the specimen onto the foil-covered sensor mat; (**c**) specimen after testing—view of the board after pressing and adhesive setting.

To protect the sensor surface, the mat was covered with a thin stretch film. A fresh adhesive dab was then applied to the center of a square EPS TR 100 façade insulation board (25 cm × 25 cm, 5 cm thick) and pressed onto the film-covered mat. The adhesive quantity was measured by weight, using portions of 200 g and 300 g of fresh mortar. The procedure is illustrated in Figure 3. The prepared specimen was manually pressed against the horizontally positioned sensor mat to a defined distance, ranging from 6 to 12 mm. Identical adhesive amounts were used within each series of samples. A new stretch film layer was applied for each adhesive dab. The test campaign included 20 adhesive dabs using a single type of cement-based adhesive for EPS insulation.

**Figure 4:**
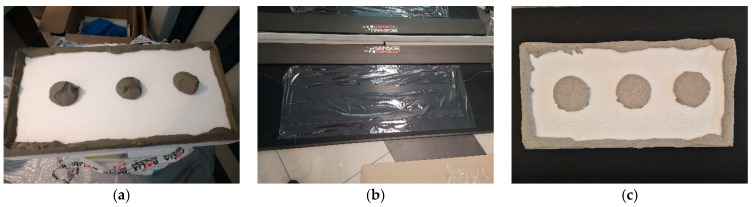
Pressure Mapping Adhesive Dab Test (PMAST)—testing of elements sized 90 cm × 40 cm: (**a**) test specimen before pressing—EPS board with a measured amount of fresh adhesive; (**b**) prepared sensor mat covered with protective film; (**c**) specimen after testing—view of the board after pressing and adhesive setting.

In addition to testing individual specimens, measurements were also carried out on two full-size EPS insulation boards, with adhesive applied using the ribbon-and-dab method. Due to the 50 cm active width of the sensor mat, EPS boards measuring 90 cm in length, 40 cm in width, and 10 cm in thickness were used. The thickness was increased compared to the 25 cm × 25 cm samples to ensure adequate stiffness of the insulation during pressing. Fresh adhesive mortar with a mass of 2.6 kg was applied to each board, following a layout consisting of a continuous ribbon along the perimeter and three dabs in the central area. The setup is shown in Figure 4.

A cement-based adhesive mortar for bonding EPS, being a component of the thermal insulation system for which a Technical Assessment was issued, was used for the test. Bulk density of the mortar: 1.44 g/cm^3^, water-to-mortar ratio: 0.20 L/kg. Approximate composition of the mortar: dry mixture of cement binder, polymers, fine-grained mineral fillers, and modifying additives. The mortar was prepared according to the manufacturer’s recommendation.

#### 2.2.3. Pull-Off Testing Using the Original CAST and DAST Methods

The objective of the testing was to assess the adhesion within specific areas of the contact surface formed by a cement-based adhesive applied as a single dab. The aim was to determine how the pressing force applied during thermal insulation installation influences the adhesive bond between the substrate and the insulation, and how the adhesive layer thickness affects adhesion to the substrate.

The tests were conducted using two original pull-off methods developed by Paweł Gaciek: CAST (Central Adhesive Spot Test) [36] and DAST (Dual-Adhesive Spot Test) [37].

The test specimens consisted of adhesive dabs with fresh mortar masses of 200 g and 400 g. A total of 12 samples were prepared: 6 with a thickness of 10 mm (200 g of fresh mortar) and 6 with a thickness of 20 mm (400 g of fresh mortar). The adhesive was applied to the central part of EPS TR 100 boards measuring 20 cm × 20 cm and 5 cm thick, and then bonded horizontally to the substrate. The substrate consisted of smooth concrete slabs with a tensile strength above 1.8 MPa, a density of 2247 kg/m^3^, and a water absorption of 28.1 g/(m^2^∙s^0.5^), meeting the requirements of EN 772-11:2011 [38]. Two different cement-based adhesives for EPS bonding were used in the tests. Both adhesives were prepared according to the manufacturers’ guidelines. The amount of mixing water was taken as the midpoint of the ranges specified by the manufacturers. Each adhesive was part of a complete ETICS system with a valid Technical Assessment.

**Figure 5:**
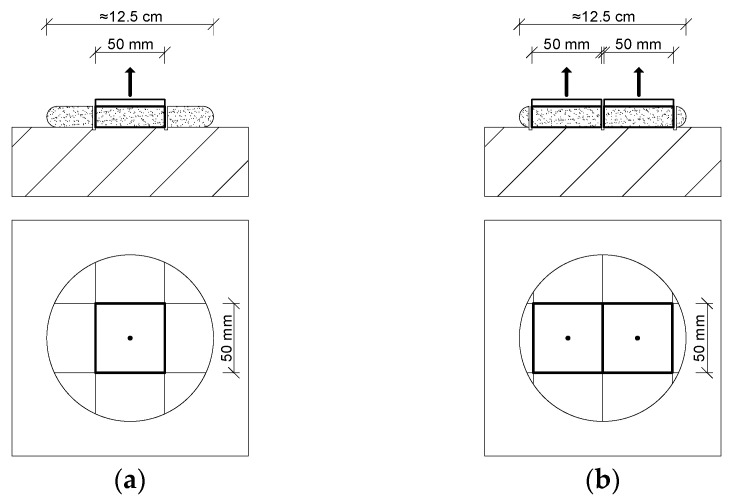
Layout of the square sections being cut from a round adhesive dab and pulled off from the concrete substrate: (**a**) one 50 mm × 50 mm section (for the CAST method); (**b**) two 50 mm × 50 mm sections (for the DAST method).

The tests were carried out in a controlled environment with a constant temperature of 23 ± 0.5 °C and a relative humidity of 50–55%. EPS boards with applied adhesive were pressed manually against the substrate to a predefined distance. After 28 days, the insulation was removed from the adhesive dabs. For three samples with adhesive layer thicknesses of 10 mm and 20 mm, square sections measuring 50 mm × 50 mm were cut from the central part of the adhesive dab (Figure 5a). For the other three samples with 10 mm and 20 mm dabs, two square sections of the same size were cut from the dab, aligned such that their edges coincided with the diameter of the adhesive spot (Figure 5b).

### 2.3. Numerical Modeling

Based on the pressure maps obtained from sensor mat measurements (Section 2.2.2), and guided by the geometric characteristics of the adhesive-substrate interface investigated in the custom adhesion tests (Section 2.2.3), a numerical model was developed to simulate the distribution of pressure beneath adhesive mortar dabs under varying loading conditions.

Complementing the real-time visualization on the baropodometric platform, this experiment also yielded numerical data on a 5 mm × 5 mm grid. These data, normalized relative to the maximum pressure value, were directly used to calculate the average pressure exerted on the substitute substrate (sensor mat), as well as to determine pressure values within predefined intervals ranging from near-zero to one. The normalized data were also used to generate interpolated maps in Surfer version 20.4.3 [39], using Delaunay triangulation with linear interpolation. This method was selected due to the uniform distribution of data points across the grid and its strict honoring of the measured values.

To further illustrate the implications of the observed pressure distributions, two test samples were selected for in-depth analysis: one with a more centralized pressure distribution, and the other exhibiting a more irregular pattern, as recorded by the sensor mats.

#### 2.3.1. Pressure Decay Function

The resulting maps were used to determine both the average pressure and the pressure values within selected zones. Furthermore, the maps served to derive a pressure decay function from the center of the dab toward its periphery. To this end, 24 radial cross-sections were defined from the geometric center of each map at 15° intervals, with data points spaced every 0.5 mm. In each cross-section, if multiple grid points exhibited a normalized value of 1, only the outermost one (i.e., the one furthest from the center) was selected for further analysis. From that point, the segment of the cross-section extending to the outer edge (value 0) was extracted. These segments were then individually normalized with respect to their total lengths, such that each segment was scaled to a unit length. Although the spacing between points within each normalized segment remained uniform, it could vary between cross-sections.

As a result of this procedure, two datasets were obtained: a primary one containing all available points (4725 in total), and a reduced one in which, for each cross-section, the starting and ending points were retained along with every tenth point from the remaining data—yielding a total of 582 points. The reduced dataset was created solely to enhance the clarity of graphical representation. The complete dataset was then used to determine the average pressure decay function, defined from a constant value of 1 at the inner boundary to 0 at the periphery.

To define the pressure decay function, four boundary conditions were imposed: *f*(0) = 1, *f*(1) = 0, *f*′(0) = 0, and *f*′(1) = 0. The latter two ensure horizontal tangents at the endpoints, enabling smooth transitions to adjacent constant-pressure segments. For simplicity and ease of integration, a double-power function was adopted, defined as:

(1) $$f\left(r\right)=\;{\left[1-w\;\cdot \;r^{n}-\;\left(1-w\right)\;\cdot \;r^{\left(k\;\cdot \;n\right)}\right]}^{2}$$

Here, *w* is a weight parameter controlling the relative contribution of the two power terms, *n* determines the overall steepness and curvature, and *k* introduces asymmetry by shifting the secondary decay component, resulting in a smooth profile and analytically tractable expression.

The parameters *k*, *n*, and *w* were estimated using the L-BFGS-B algorithm—a constrained variant of BFGS that supports parameter bounds. To ensure a seamless connection with the constant parts of the piecewise-defined function, a penalty term was added to the objective function to suppress the slope at *r* = 0, the start of the decay segment.

L-BFGS-B is a gradient-based quasi-Newton optimization method that uses first-order derivative information, either supplied analytically or estimated numerically. The procedure was executed using the optim() function in the R environment [40], within the RStudio IDE (Integrated Development Environment) [41].

#### 2.3.2. Simulation of Pressure Distribution

After establishing the pressure decay function, a simulation model was developed in the R environment to replicate pressure distributions beneath circular mortar dabs under varying boundary conditions and stochastic perturbations.

**Figure 6:**
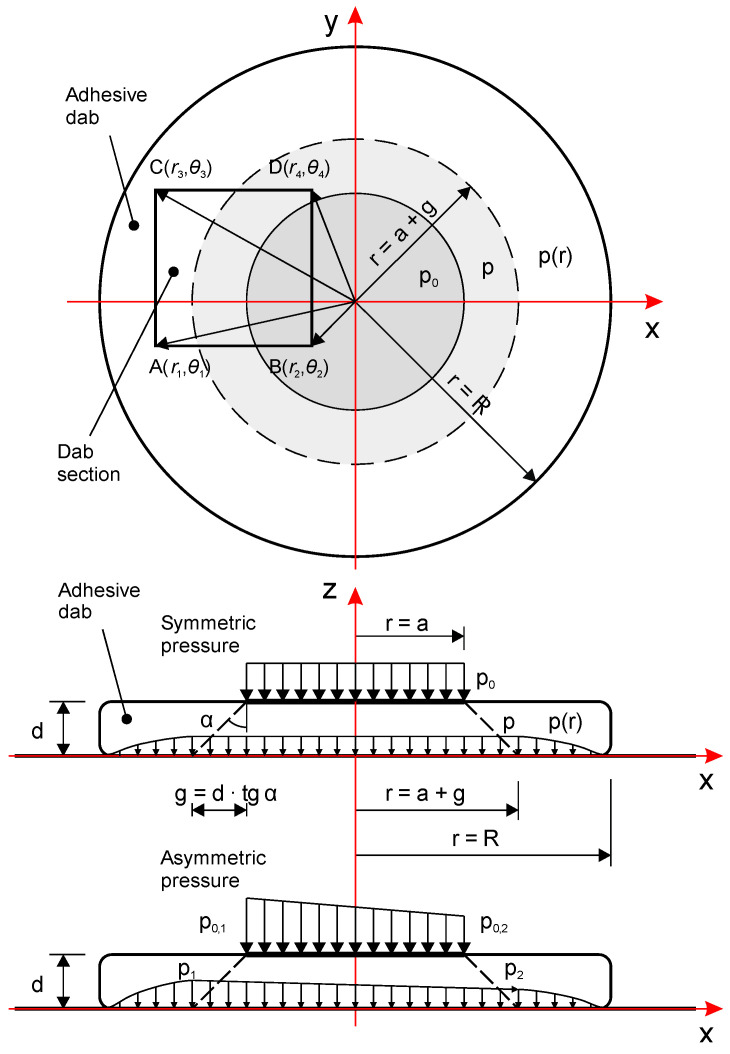
Schematic diagram of the pressure distribution model in an adhesive mortar dab. The distribution of pressure does not include irregularities introduced by Perlin noise.

In the numerical model whose scheme is shown in Figure 6, a circular adhesive mortar dab with radius *R* and thickness *d* is considered. A vertical load is applied to its top surface over a circular area of radius *r* = *a*, where *a* is treated as a random variable within defined bounds. To account for possible asymmetry in the pressure application, the model includes a linear modification of the external pressure depending on the *x* and *y* coordinates. This variability is defined using a linear function controlled by coefficients *k*_x_ and *k*_y_, whose values are also randomly assigned. The applied pressure *p*_0_ is then assumed to spread linearly through the adhesive layer at an angle *α*. As a result, the contact area on the bottom surface (i.e., the interface with the substrate) increases, forming a circular area with radius *r* = *a* + *g*, where *g* = *d*·tan(*α*). This expansion leads to a proportional reduction in pressure intensity to value *p*.

The adopted angle *α* was also used to determine the parameter *a* value in the range resulting from the images obtained on the sensor mats, moving upwards from the area with maximum pressure on the lower surface.

To ensure easy comparability, the total force was scaled so that the maximum pressure at the bottom surface (for *d* = 10 mm) equals 1.

From the final point (circle) of linear propagation of pressures, the function of their disappearance towards the edge of the mortar dab begins. The decay ends at a defined cutoff point from the edge, forming a peripheral ring where pressure is zero or below detection threshold—a feature observed in the experiments (Section 2.2.3).

Consequently, the modeled radial pressure function comprises three segments:

- A constant central region (value = 1);
- A nonlinear decay zone (described by the double-power function);
- A constant outer ring (value = 0).

To simulate the irregular adhesive-substrate contact caused by surface roughness or load variability, Perlin gradient noise [42] was added to the pressure distribution. The noise parameters—frequency, amplitude, and reference level—were randomized. The noise was scaled relative to the maximum pressure value and mortar thickness. Any pressure values exceeding the maximum (due to noise or linear modification) were clipped to the upper bound.

The simulation and visualizations were implemented in R, using libraries such as ambient [43], akima [44], dplyr [45], and ggplot2 [46]. For any selected rectangular or square region (denoted ABCD)—covering all or part of the adhesive dab—the model outputs:

- The specific random parameter values used;
- Minimum, maximum, and mean pressure values;
- The surface areas and corresponding proportions falling within predefined pressure intervals (ranging from 0 to 1).

To evaluate the influence of model parameters on the resulting pressure distribution, a Monte Carlo simulation was carried out. A total of 10,000 randomized runs were performed for each of 10 independent random seeds, resulting in 100,000 model realizations. In each run, values for six key parameters were sampled independently: the loading radius *a*, the pressure unevenness coefficients *k*_x_ and *k*_y_, and three Perlin noise parameters—frequency, amplitude, and base level. For each simulation, the resulting normalized pressure field was computed and classified into predefined pressure intervals, enabling direct comparison with interpolated empirical pressure maps. The model outputs were evaluated based on the surface area shares within each interval and the average pressure values.

The initial parameter intervals, selected based on qualitative assessment, were subsequently refined through analysis of the best-fitting configurations identified in the Monte Carlo simulations. A second full-scale Monte Carlo simulation was then performed using the narrowed parameter ranges, resulting in improved alignment with empirical observations.

The current pressure distribution model can be classified as quasi-geometric with empirical calibration. It does not explicitly account for the rheological or elastic properties of the adhesive mortar and is not intended to replicate material deformation behavior.

Planned extensions of the model include the incorporation of a logistic (sigmoidal) function, which, once empirically calibrated, will allow for the derivation of bond strength maps at the adhesive-substrate interface.

The R codes used for numerical modeling are provided as Supplementary Materials. The optimization of these codes and selected text refinement were supported by generative artificial intelligence (GenAI) tools.

## 3. Results and Discussion

### 3.1. In Situ Investigations

**Figure 7:**
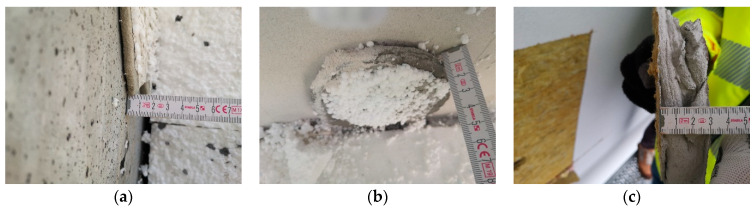
Example measurements of adhesive mortar thickness between the insulation and the substrate: (**a**) 7 mm thick adhesive dab; (**b**) 20 mm thick adhesive dab; (**c**) 30 mm thick adhesive dab.

Cross-sectional field inspections revealed that the thickness of the adhesive layer bonding the thermal insulation to the wall most commonly ranged from 7 to 20 mm (Figure 7). Relatively frequently, adhesive layers exceeding 20 mm were recorded, occasionally reaching 30 mm, and in isolated cases up to 40 mm. Greater thicknesses were typically associated with damaged ETICS systems and were not observed at any meaningful scale in standard construction practice. A frequent and highly significant finding from the in situ inspections—critical for the effectiveness of adhesive bonding—is the variability in adhesion between the adhesive mortar and the substrate. This is typically caused by technical deficiencies of the substrate, such as its poor condition or inadequate preparation prior to insulation installation. In cases where delamination occurs within the substrate layers (cohesion fracture in the substrate material) or when the adhesive detaches from the substrate while pulling off fragments of it (predominantly adhesion fracture with partial cohesion fracture in the substrate, i.e., with substrate fragments attached), the cause is relatively straightforward to identify. However, another commonly observed phenomenon is the variation in adhesion strength across different areas of a single adhesive dab on an insulation board, even when the substrate condition is uniform. In such cases, detachment occurs solely at the adhesive–substrate interface and is classified as a pure adhesion fracture.

**Figure 8:**
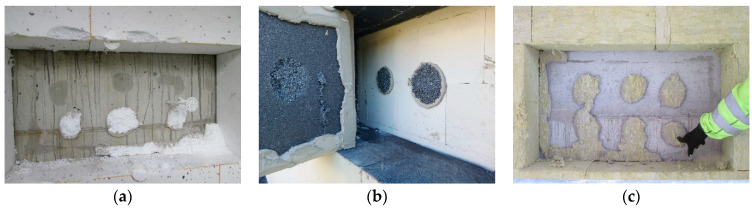
Partial detachment of adhesive mortar from substrates with visually uniform surface properties: (**a**) reinforced concrete wall with white EPS thermal insulation (TR 100); (**b**) wall made of calcium silicate blocks with graphite EPS insulation (TR 100); (**c**) reinforced concrete wall with mineral wool insulation (standard façade board, TR 10).

Figure 8a shows that the insulation boards were bonded using the ribbon-and-dab method. During the removal test, part of the hardened adhesive mortar detached from the substrate and remained adhered to the insulation, leaving visible traces on the substrate surface. The other part remained bonded to the substrate, with fragments of the thermal insulation torn off and left on the adhesive. Figure 8b shows a case in which the perimeter ribbon of adhesive completely detached from the substrate, while the central dabs remained adhered to the wall surface with torn EPS residues attached—indicating bond strength exceeding the tensile strength of the insulation, despite the removal not being perfectly perpendicular. Figure 8c shows a case where all adhesive dabs remained bonded to the structural substrate surface. The perimeter adhesive partially detached from the substrate and remained on the surface of the mineral wool, despite the fact that the tensile strength of façade-grade mineral wool (TR 10) is significantly lower than that of EPS (TR 100).

**Figure 9:**
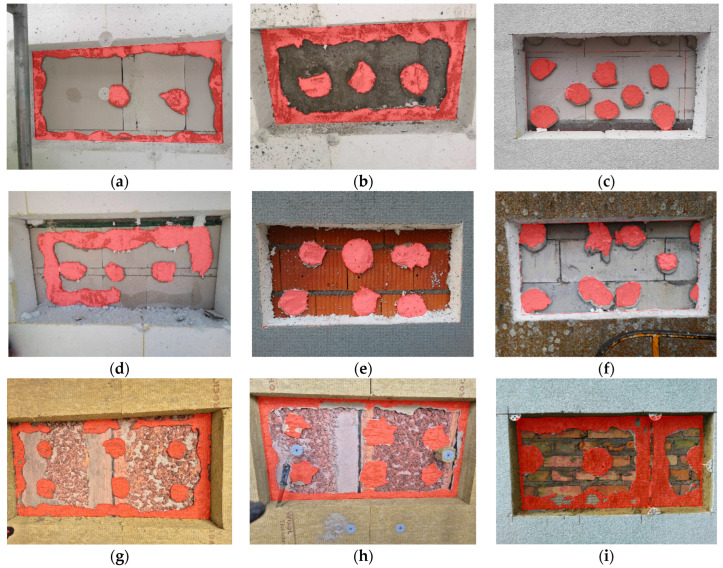
Examples of insulation detachment from the wall with computer-generated red overlays indicating areas of effective bonding between the adhesive mortar and the insulation: (**a**–**f**) expanded polystyrene (EPS); (**g**–**i**) mineral wool (MW).

Figure 9 presents selected examples of thermal insulation detachment from walls, with a computer-generated red overlay indicating the area of effective bonding between the adhesive mortar and the insulation. The EPS boards measured 50 cm × 100 cm, while the mineral wool boards measured 60 cm × 120 cm. The results, expressed as the percentage of the bonded surface area, are summarized in Table 1.

The assessment of the effective bonding area between the thermal insulation and the adhesive layer indicates that only in the case of two boards was the minimum requirement in Poland met—boards (b) and (i). The adhesion condition of boards (c), (e), and (f) does not meet either the requirement of the minimum effective bonding area or the requirement of proper adhesive distribution on the board (absence of a perimeter adhesive ribbon).

**Figure 10:**
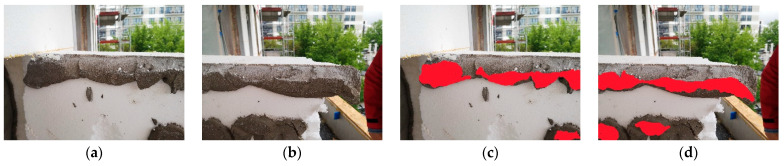
View of a representative thermal insulation board detached from a concrete substrate: (**a**,**b**) actual view after detachment; (**c**,**d**) red-marked contact area of the perimeter adhesive ribbon with the substrate, highlighting the difference between the contact area with the substrate and the bonded area with the EPS board.

The conducted investigations revealed that increasing the thickness of the adhesive layer generally leads to a reduction in the effective contact area with the substrate—particularly within the perimeter ribbon of the adhesive. Observations also indicate that the perimeter ribbon develops a proper and continuous shape only when the adhesive layer remains relatively thin, typically below 15 mm, provided that a sufficient amount of mortar is applied to ensure effective bonding of the insulation. A proper ribbon shape can be defined as one with a minimum width of 50 mm. However, even at this width, sufficient adhesion to the substrate may not be ensured. A key influencing factor is the pressing force applied to the insulation board during installation, which affects both the spread of the adhesive between the insulation and the substrate, and the peak contact pressure. When the adhesive layer is relatively thick, the perimeter ribbon often fails to develop a regular shape—similar irregularities are also observed in the central adhesive dab. As a result, it becomes difficult to achieve the minimum required contact area with the substrate and to generate enough pressure to ensure adequate adhesive bond strength. Due to the fact that the adhesive mortar is typically applied to the insulation board, it establishes an initial, partially forced contact with it. The application technique and the tools used ensure this preliminary adhesion between the mortar and the insulation surface. In the case of EPS boards, the adhesive dabs are applied by throwing the mortar onto the board with a trowel, and in some areas additionally rubbing it in. The perimeter ribbon is applied in a way that subjects the mortar to considerable tool pressure, especially when forming the characteristic prism shape along the board’s edges. As a result, the adhesive is sufficiently bonded to the EPS board to prevent it from falling off when the board is moved from a horizontal to a vertical position and then pressed onto the wall. For mineral wool, standard practice involves an initial application of a thin layer of mortar rubbed into the wool’s surface to improve adhesion (referred to in the industry as a contact layer). In the next step, using the “wet-on-wet” technique, this layer is combined with the main layer of adhesive, which forms both the perimeter ribbon and central dabs. This approach eliminates problems related to the limited adhesion of the mortar to mineral wool, as the pressing force applied during installation provides additional support to the bond between the adhesive and the insulation board. In contrast, when it comes to the contact between the adhesive mortar and the substrate, the pressing force applied during installation is the sole factor influencing adhesion. This aspect becomes particularly important in cases involving thicker adhesive layers. In such situations, the contact area between the mortar and the insulation is significantly larger than the contact area with the substrate, which results from the lower effective pressing force. This phenomenon is confirmed by the results of the in situ inspections (Figure 10).

In the analyzed cases, the contact area between the adhesive and the insulation was up to 50% greater than the corresponding contact area with the wall substrate.

**Figure 11:**
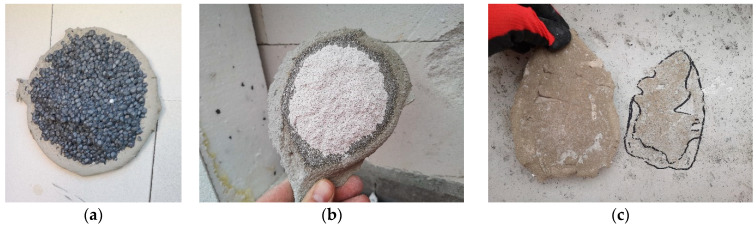
Presence of zones with varying adhesion of adhesive dabs to insulation and substrate observed during insulation removal inspections: (**a**) detachment of graphite EPS from the adhesive dab; (**b**) detachment of the adhesive dab from an autoclaved aerated concrete block substrate (cohesion fracture in the substrate); (**c**) detachment of the adhesive dab from formwork-cast concrete.

When analyzing the results of insulation removal during in situ inspections, zones and areas were also identified within the adhesive dabs where adhesion to either the substrate or the thermal insulation was insufficient or entirely absent. This was observed on the side of the bond between the adhesive mortar and the insulation layer (Figure 11a), as well as on the side of the substrate, which in this case was made of autoclaved aerated concrete (Figure 11b) or regular concrete. In the latter, black lines were used to mark the actual bonded area of the adhesive dab on the substrate surface to allow for comparison with the actual dimensions of the mechanically detached mortar dab (Figure 11c).

### 3.2. Contact Surface Analysis Using the GCAT Method

**Figure 12:**
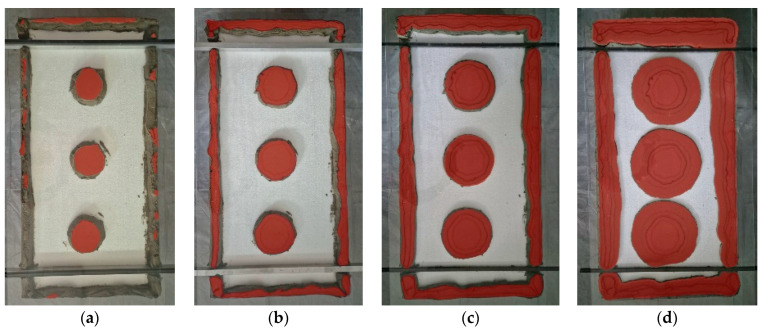
View of the adhesive contact surface with the substitute substrate for varying adhesive layer thicknesses: (**a**) 30 mm; (**b**) 20 mm; (**c**) 15 mm; (**d**) 10 mm.

The original GCAT (Glass Contact Area Test) method enabled the identification of the actual effective contact area between the adhesive mortar and a substitute substrate—specifically, a glass pane placed against the mortar with the aid of spacers to maintain a defined adhesive layer thickness (Figure 12). A total of 6 kg of fresh adhesive mortar was used to bond a single thermal insulation board. The adhesive mass was experimentally predetermined to demonstrate a specific effect.

The analysis shows that at the greatest adhesive layer thickness of 30 mm, the perimeter adhesive ribbon barely engages with the substrate, rendering the measurement of its width irrelevant. Full contact between the thermal insulation and the substrate is provided exclusively by adhesive dabs, which are geometrically close to circles with diameters of 104–105 mm. However, even this results in a contact area significantly below the minimum requirement [26]. At an adhesive thickness of 20 mm, a continuous perimeter ribbon becomes visible, with a width of 22–33 mm. The diameter of the adhesive dabs increases to 136–144 mm. Applying a 15 mm thick layer results in a further increase in the width of the perimeter ribbon, reaching 40–55 mm. The diameters of the adhesive dabs in this case range from 172 to 178 mm. With a 10 mm thick adhesive layer, a substantial increase in the contact area with the substrate is observed. The width of the perimeter ribbon reaches 60–75 mm, and the diameter of the adhesive dabs increases to 235–241 mm.

Table 2 summarizes the contact areas between the adhesive mortar and the substitute substrate for different adhesive layer thicknesses, including the total contact area as well as the separate contributions from the perimeter ribbon and the dabs. At a thickness of 30 mm, there is almost no contact between the perimeter ribbon and the substrate, and the total adhesive–substrate contact area—concentrated almost entirely in the dabs—does not exceed 8% of the insulation board’s surface. For a 20 mm adhesive layer, the contact area reaches approximately 24%, which meets the minimum requirement specified in [26], but falls short of national requirements [25,27] and the conditions stated within technical approvals. Compared to the 30 mm case, the perimeter ribbon begins to play a noticeable role in bonding to the substrate. Only at a 15 mm adhesive thickness does the contact area approach 40%, which is considered the minimum recommended bonding area for adhesively fixed ETICS systems in Poland. In the 10 mm variant, the contribution of the perimeter ribbon to the effective bonding area increases substantially. In this case, the perimeter ribbon alone accounts for nearly 40% of the total contact area. This configuration results in a total contact area that is nearly 40 percentage points higher than with the 20 mm layer, and more than 56 percentage points greater than that observed with the 30 mm layer. It is also worth noting that the share of the contact area contributed by the adhesive dabs, relative to the total contact area, ranged from approximately 37% to 42% for adhesive layer thicknesses of 10, 15, and 20 mm.

### 3.3. Pressure-Sensing Mat Tests Using PMAST Method

The testing method involving a baropodometric platform, as described earlier, enabled the visualization of the pressure distribution of the adhesive mortar on a substitute substrate (sensor mat). A key to interpreting the applicability of the results from such a test is being aware of its dynamic nature and relating the observed effects to the actual process of bonding the thermal insulation board to the substrate.

On the one hand, the sensor mat allows for the measurement of specific force values with which the adhesive mortar is pressed against the substrate through the thermal insulation element. On the other hand, it is important to note that these forces result from manual pressure, and their magnitude and variation are specific to this single, non-repeatable test case. Reproducing the action with exactly the same force is practically impossible and inherently random, yet it closely resembles real-world application conditions.

The pressure transmission mechanism itself—the core of the study—remains fully repeatable. The resulting visualizations, presented as color maps, illustrate the variation in pressure values across different areas of a single mortar dab. The accuracy of pressure differentiation naturally depends on the number and sensitivity of sensors per unit area, the interpolation algorithm used in the software, and the settings of the color scale on the map.

In the study, the samples were pressed manually in a manner similar to the actual application of thermal insulation boards. The resulting force depends on the amount of adhesive used, the rate of displacement of the board relative to the substrate (sensor mat surface) during pressing, and the final gap between the board and the substrate, which determines the thickness of the adhesive layer. During the dynamic course of the test, as the gap between the insulation and the substitute substrate decreases, the pressure increases, but at the same time, the area of interaction increases due to the spreading of the mortar across the mat and beneath the insulation. This continues until the adhesive reaches a final thickness—either intended or incidental. The forces reach their peak values at various locations within the mortar dab and diminish as the manual pressing ends.

**Figure 13:**
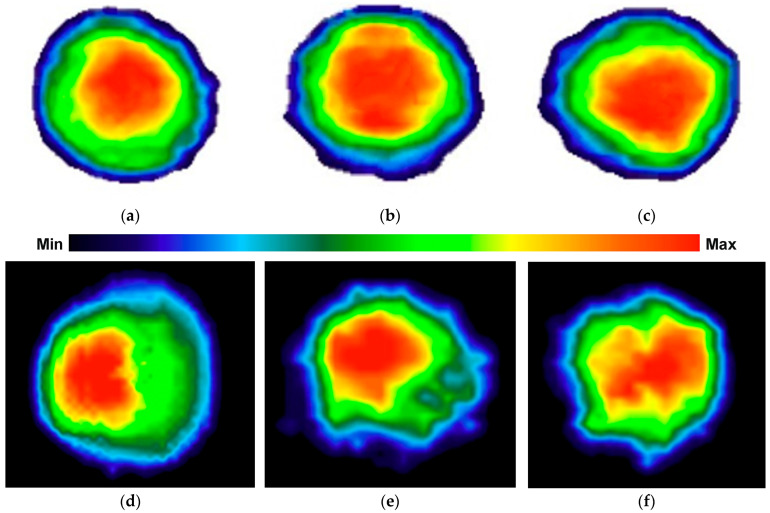
Color maps illustrating the pressure distribution in adhesive dabs made from the same adhesive mixture with identical weight and consistency: (**a**–**c**) adhesive evenly distributed in the shape of a truncated cone and pressed almost perpendicularly; (**d**,**e**) angled pressing, which shifts the area of maximum pressure and results in uneven adhesive layer thickness; (**f**) nearly perpendicular pressing with irregular adhesive distribution on the sample surface.

The color maps indicate the peak contact force values in individual surface regions where the adhesive spreads during the pressing process, simulating actual adhesion to the substrate. In addition to pressure distribution patterns, an important factor in such testing is the adhesive mortar itself, particularly its consistency and rheological behavior, as they influence both the intensity and distribution of contact forces across the sensor mat. Examples of color maps for several dabs made from the same adhesive mixture with identical weight and consistency are shown in Figure 13. The color maps clearly show that the highest pressure consistently occurs in the central region of each mortar dab. Some displacement of this area depends on the distribution of the adhesive during application and the direction of the manual pressing force.

In the case of an adhesive mortar dab, the area in which the adhesive spreads under pressure is not laterally constrained—it is confined only by the horizontal planes: by the sensor mat at the bottom and the insulation board at the top. As the pressing force reaches its peak in the central region, it decreases radially outward and fades toward the edges.

Figure 13a–c show the results of pressing samples with evenly distributed adhesive shaped as a truncated cone, using an almost perpendicular pressing force. Figure 13d,e present the effects of angled pressing force, which shifts the area of maximum pressure and leads to an uneven thickness of the mortar layer. Figure 13f illustrates pressing performed nearly perpendicularly, but with irregular adhesive distribution on the surface of the sample.

**Figure 14:**
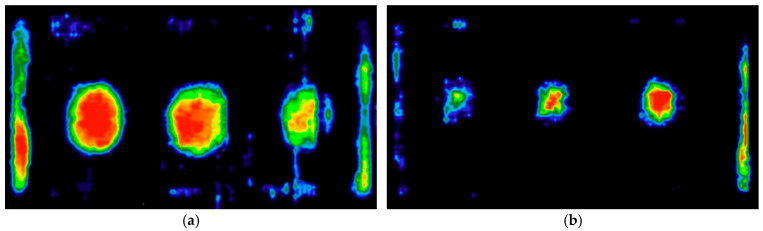
Color maps illustrating the pressure distribution during bonding of a thermal insulation board with an adhesive layer thickness of (**a**) 8 mm; (**b**) 11 mm.

Testing the “bonding” of a full thermal insulation board to the substitute substrate (sensor mat) proved more complex, as it is difficult to manually press the entire board in a single operation that accurately replicates real-world application conditions. Example pressure distributions during board bonding with varying, random adhesive layer thicknesses are shown in Figure 14.

The color maps indicate that maximum pressures still occur in the central parts of the mortar dabs, with only limited occurrence in the peripheral zone (Figure 14a,b). Most of this edge region was not even registered by the sensors due to the low pressure present in those areas. The issue of insufficient pressure is particularly evident when bonding a full board, where a thicker adhesive layer was observed despite using the same amount of material—as illustrated in Figure 14b.

### 3.4. Pull-Off Tests Using the CAST and DAST Methods

The areas of varying pressure within a single adhesive dab, as observed in the pressure-sensing mat tests, were verified using pull-off testing with two different methods. Table 3 presents the results of pull-off tests performed on samples detached from the substrate: from the center of the dabs (labeled “center”) with adhesive layer thicknesses of 10 mm and 20 mm, as well as from the left and right halves of the dabs (labeled “L” and “R”), positioned symmetrically relative to the dab’s central axis. The tests were conducted using two different cement-based adhesives for bonding EPS boards.

**Figure 15:**
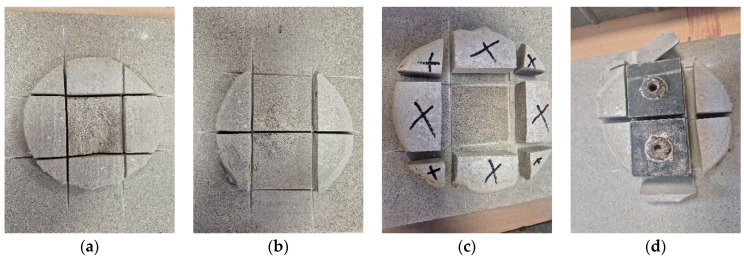
Representative images of adhesive dabs being detached from the concrete substrate using a pull-off testing device: (**a**) dab with a thickness of 10 mm—detachment of a single sample from the geometric center of the dab; (**b**) dab with a thickness of 10 mm—detachment of two samples with one common edge aligned along the dab’s diameter; (**c**) dab with a thickness of 20 mm—detachment of a single sample from the geometric center of the dab; (**d**) dab with a thickness of 20 mm—detachment of two samples with one common edge aligned along the dab’s diameter.

The detachment patterns of the tested samples shown in Figure 15a,b are quite characteristic. Specifically, for adhesive dabs with a 10 mm layer thickness, samples pulled from the central region exhibited cohesive failure within the adhesive layer, accompanied by relatively high pull-off force values. In contrast, samples taken symmetrically from the left and right sides of the same dab (Figure 15b) showed mixed adhesive–cohesive failure: the cohesive portion was located near the center of the dab, while adhesive failure occurred on the outer edges of both samples. This provides compelling evidence of varying adhesion zones within a single adhesive dab, corresponding to the pressure distribution patterns previously observed using the sensor mats.

Pull-off tests performed on dabs with a 20 mm adhesive thickness yielded significantly lower values and consistently showed purely adhesive failure at the interface between the substrate and the adhesive. Moreover, during sample preparation, some peripheral parts of the dab detached spontaneously due to extremely weak bonding. These fragments are marked with black crosses in Figure 15c and were located at the outer perimeter of the dab—further confirming weaker adhesive bonding in areas with reduced pressure during application.

In all cases, the pull-off force values for the two symmetrical samples were markedly lower than for the sample taken from the center of the dab. This applies to both 10 mm and 20 mm adhesive thicknesses. Furthermore, pull-off strength in the 10 mm variant was consistently several times higher than that recorded for the 20 mm dabs. This pattern was observed for both adhesive types.

It can therefore be concluded that the highest adhesion to the substrate occurs in the central region of the dab, where the pressure during the bonding phase was the greatest. Increasing the adhesive layer thickness—while proportionally increasing the amount of adhesive—generally leads to a significant reduction in the overall adhesion of the dab to the substrate. However, the internal pattern of adhesion variation within the dab remains consistent.

**Figure 16:**
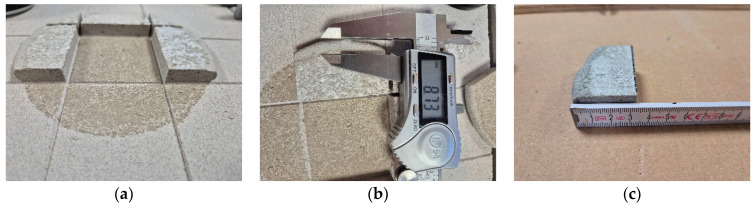
Partially detached fragments of the adhesive dab from the substrate, revealing a low-adhesion zone at the interface between the dab and the substrate: (**a**) substrate surface with remnants of the adhesive dab after the CAST test; (**b**,**c**) measurement of the zero-contact ring width.

Further insight into these findings was provided by a visual assessment and analysis of the perimeter zone of a representative adhesive dab with a thickness of 10 mm and an approximate diameter of 125 mm (average of 10 measurements). The dab was applied using the original CAST method. After 28 days of curing under laboratory conditions, the insulation board was removed from the surface, and sections of the dab were peeled away to reveal the actual appearance of the outer perimeter zone that showed no contact with either the substrate or the insulation (Figure 16). In this particular case, the non-contact zones on both sides were similar in extent and accounted for just over 14% of the total projected area of the dab. This estimate does not include the adjacent inner zone exhibiting low adhesion.

Consequently, in real-world ETICS applications as well as in laboratory tests, adhesion is generally absent in the peripheral edge areas of adhesive dabs. For dabs with a diameter of approximately 125 mm and thicknesses of 10 or 20 mm, the width of the zone near the edge exhibiting no or significantly reduced adhesion was estimated at 7–15 mm, depending on the dab’s thickness, diameter, and the consistency of the adhesive mortar.

### 3.5. Results of Numerical Analyses

#### 3.5.1. Pressure Maps

**Figure 17:**
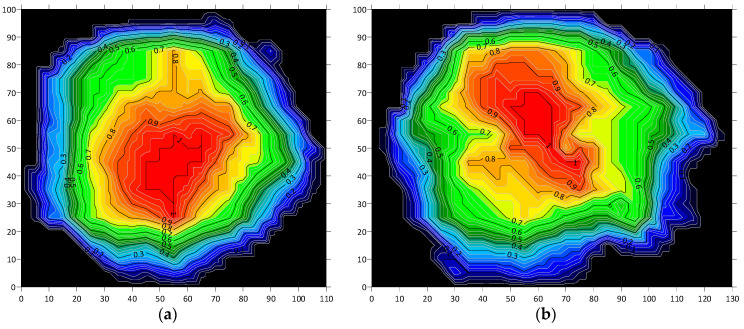
Contour plots of the normalized pressure maps obtained using triangulation with linear interpolation: (**a**) Sample 2; (**b**) Sample 6. Colors indicate normalized pressure values, with black contour lines plotted at 0.1 intervals and gray contour lines at 0.025 intervals. Dimensions of maps are given in millimeters.

As part of the numerical analysis, two test samples from the sensor mat experiments—denoted as Sample 2 and Sample 6—were selected for detailed examination. Initially, normalized pressure maps were generated to represent the contact surface between the adhesive mortar dabs and the substitute substrate. These maps were obtained through triangulation with linear interpolation and are presented in Figure 17, as contour plots.

Table 4 and Table 5 summarize the normalized pressure distributions and average values for two test samples. For each case, both the raw 5 mm × 5 mm grid data and the interpolated maps (generated in Surfer) were analyzed to compare surface pressure distributions.

The results of the pressure distribution analysis beneath two adhesive mortar dabs (referred to as Sample 2 and Sample 6) are presented in Table 4. The surface area shares corresponding to specific pressure intervals were estimated using two approaches: (1) based on values assigned to a regular 5 × 5 mm grid, reflecting the spatial arrangement of data collection by the pressure-sensing mat, and (2) using interpolated pressure maps generated with Surfer software. In both approaches, pressure values were normalized relative to the maximum recorded value.

Table 5 compares the geometric and average pressure values obtained by both methods for each sample. The average pressures tend to be slightly lower in the interpolated maps, which may result from smoothing effects introduced during interpolation. Despite geometric differences between the samples (Sample 6 featured a more elongated and oblique contact area), the pressure distributions demonstrate consistent patterns, supporting the robustness of the analytical methodology.

Additionally, Table 6 presents the mean normalized pressure values within 0.05 m × 0.05 m sections of the adhesive mortar dab. Five section positions were considered: center, left, right, top, and bottom—the latter two accounting for the variability in mortar placement relative to the reference frame.

Assuming the mean pressure in the central section as the reference (100%), the mean pressure over the entire mortar dab corresponds to 64.08% and 55.26% for Sample 2, and 65.58% and 56.93% for Sample 6, based on the 5 mm × 5 mm grid and the interpolated maps, respectively. The grand mean of these values is 60.46%.

#### 3.5.2. Fit of the Pressure Decay Function

As a result of the optimization procedure, the parameters of the double-power function (Equation (1)) were estimated as follows: *w* = 0.5, *k* = 4.8, and *n* = 1.6, yielding the final analytical form:

(2) $$f\left(r\right)=\;{\left(1-0.5\;\cdot \;r^{1.6}-\;0.5\;\cdot \;r^{7.68}\right)}^{2}$$

This expression corresponds to the middle segment of the piecewise-defined pressure function, discussed further in Section 3.5.3.

**Figure 18:**
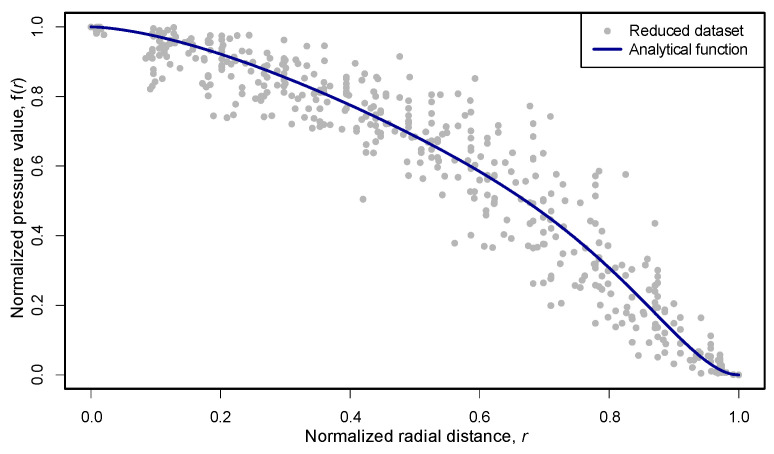
Comparison of the fitted analytical pressure decay function and the reduced dataset.

The fitted parameter values indicate a balanced contribution of the two power terms and a moderately steep, asymmetric decay. The relatively high value of *k* shifts the secondary decay component closer to the periphery, capturing the experimentally observed delay in pressure reduction in both samples. The fitted function *f*(*r*) plotted against the reduced dataset is shown in Figure 18.

The quality of the fit was assessed using the coefficient of determination. For the complete dataset, the value obtained was *R*^2^ = 0.928, and for the reduced (sparse) dataset, it was *R*^2^ = 0.954. The agreement between the model and the empirical data is therefore considered to be very good.

In addition to the determination coefficient, further error metrics confirmed the high quality of the fit. For the full dataset, the standard error of estimate was 0.0833 and the mean absolute deviation (MAD) was 0.0606. In the reduced dataset, these values improved to 0.0760 and 0.0495, respectively. The maximum deviation in both datasets was 0.3086. These results confirm that the model provides an accurate and robust approximation of the empirical decay profile.

#### 3.5.3. Numerical Simulation Model

Building upon the numerical framework described in Section 2.3, the calibrated decay function (Equation (2)) was incorporated into the full simulation model to compute pressure distributions beneath adhesive mortar dabs under various geometric and boundary conditions.

The complete radial pressure function was defined as a piecewise function comprising three segments:

(3) $$\left\{\begin{array}{ll} 1, & for\;r\leq a+g\\ {\left[1-0.5\;\cdot \;{\left(\frac{r-\left(a+g\right)}{r_{c}-\left(a+g\right)}\right)}^{1.6}-0.5\cdot {\left(\frac{r-\left(a+g\right)}{r_{c}-\left(a+g\right)}\right)}^{7.68}\right]}^{2}, & for\;a+g<r\leq r_{c}\\ 0, & for\;\;r>r_{c} \end{array}\right.$$

where

*a* is the upper radius of the load application zone;
*g* = *d* ⋅ tan(*α*) is the radial extent of pressure spreading;
*d* is the thickness of the adhesive mortar dab;
*α* is the angle of pressure spreading in the mortar;
*r*_c_ = *R* − *δ* denotes the cutoff radius, with *δ* representing the width of the peripheral zero-pressure ring.

The resulting function reproduces a central plateau with uniform pressure, followed by a smooth nonlinear decay and eventual drop to zero near the periphery. This formulation ensures continuity and differentiability between the segments, matching the observed characteristics of the pressure maps derived from sensor mat measurements.

A scalar multiplier was also applied to rescale the pressure values such that the maximum pressure on the bottom surface reached 1.0 under reference conditions (*d* = 0.01 m).

Due to the non-repeatable nature of manual adhesive application and the spatially variable behavior of mortar during thermal insulation installation, several parameters in the numerical model were treated as stochastic. Their variability accounts for uneven pressure distributions introduced by manual pressing, as well as potential local inhomogeneities in the adhesive layer. The following parameter ranges were initially selected based on geometric constraints and qualitative alignment with experimental observations (pressure maps):

- Radius of initial loading on the upper surface: *a* ∈ [0.008, 0.011] m;
- Pressure unevenness coefficient in the x-direction: *k*_x_ ∈ [−5.00, 5.00];
- Pressure unevenness coefficient in the y-direction: *k*_y_ ∈ [−5.00, 5.00];
- Perlin noise frequency: 40.0 to 50.0;
- Perlin noise amplitude: 0.25 to 0.35;
- Perlin noise level (baseline): 0.90 to 0.95.

To evaluate the suitability of these parameter intervals and identify configurations that best reproduce the empirical distributions, an extensive Monte Carlo analysis was conducted. It consisted of 10 independent batches of 10,000 randomized model runs each, using different random seeds (from 123 to 132 in R environment), totaling 100,000 simulations. Each simulation yielded a normalized pressure field within the mortar contact zone, which was classified into predefined pressure intervals. The resulting percentage distributions were compared with the interpolated experimental maps for Sample 2 and Sample 6 (see Table 4). Model accuracy was assessed using the following error metrics:

- RMSE (Root Mean Square Error);
- MAE (Mean Absolute Error);
- Absolute deviation from the empirical mean normalized pressure (see Table 5).

All metrics were computed separately for Sample 2 and Sample 6. The average RMSE across both samples, denoted as mean RMSE, was used as the primary indicator of fit.

It was assumed that the mean normalized pressure values should lie within the range defined by the experimental data, including both the interpolated pressure maps and the original maps obtained directly from the sensor mat (i.e., pressure maps with a 5 × 5 mm grid). Simultaneously, the lowest possible value of mean RMSE was sought as the principal indicator of model accuracy.

In the initial analysis based on the broad parameter intervals, the resulting mean normalized pressure ranged from approximately 0.495 to 0.562. While this range was not below the value obtained from the interpolated map for Sample 6 (0.4882), it exceeded the corresponding value from the original sensor grid map for Sample 2 (0.5510), which served as a conservative upper reference. To improve consistency with physical constraints and experimental data, the parameter ranges were subsequently narrowed:

- *a* ∈ [0.008, 0.0093] m;
- *k*_x_, *k*_y_ ∈ [−4.5, 4.5];
- Amplitude ∈ [0.26, 0.35];
- Frequency and level remained unchanged.

A second full-scale Monte Carlo simulation was then performed using the refined intervals. The updated results revealed a narrower span of normalized average pressures (0.494 to 0.550), which better aligned with the empirical values and no longer exceeded those derived from the original sensor grid. The structure of the results also improved: the distribution of points in the average pressure vs. mean RMSE plot became more compact, with most simulations concentrated around average pressure ≈ 0.52 (compared to ≈ 0.53 previously) and mean RMSE ≈ 2.1. This shift suggests improved alignment of the model outcomes with the expected physical behavior of the adhesive dab.

From each simulation, the 10 best-fitting configurations (based on mean RMSE) were identified. These top-performing cases exhibited mean normalized pressures in the range of 0.5028 to 0.5259 and mean RMSE values close to 1.5, reflecting a high level of agreement with both empirical pressure maps.

RMSE is expressed in percentage points and quantifies the root mean square deviation across all pressure intervals. A value of 1.5 implies that, on average, model predictions deviate from empirical values by approximately 1.5 percentage points per interval, considering squared differences.

**Figure 19:**
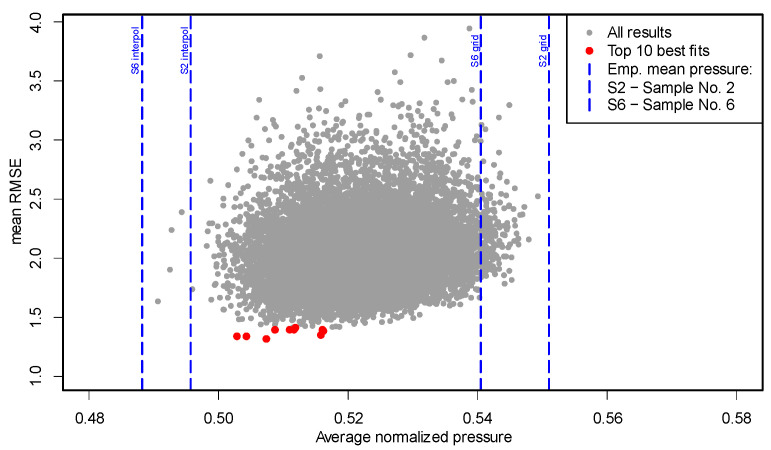
Results of the Monte Carlo simulation (10,000 runs, seed = 132). Each point represents a single run of the numerical model with a distinct set of randomly sampled parameters. The horizontal axis shows the average normalized pressure in the adhesive contact zone, while the vertical axis indicates the mean RMSE, calculated as the average error relative to the interpolated empirical maps for Sample 2 and Sample 6. Red points denote the 10 best-fitting simulations.

An example visualization based on one of the simulation sets (seed = 132 in R environment) is shown in Figure 19, illustrating the distribution of results and highlighting the best-matching parameter configurations.

Parameter sets identified within the Top 10 results of the Monte Carlo analysis can serve as a starting point for further evaluation of pressure distribution patterns. One representative case is analyzed below.

For comparison with the empirical pressure maps obtained from the sensor mat, the numerical model was configured with a mortar patch radius of *R* = 0.055 m and no peripheral zero-pressure ring (*δ* = 0). In contrast, for the numerical simulations corresponding to laboratory pull-off tests of the adhesive mortar (see 3.4), a slightly larger radius of *R* = 0.06 m was used, along with a peripheral zero-pressure zone defined as *δ* = 0.2*R*.

Two adhesive dab thicknesses were analyzed: *d* = 0.01 m and *d* = 0.02 m. In addition to the full circular dab, square sections of 0.05 × 0.05 m were extracted for local analysis. These included symmetric areas located on both sides of the vertical axis as well as a central square zone (see Figure 5 for reference). Since the orientation of the mortar dab is arbitrary in the numerical model, local analysis was also performed on square sections extracted from the top and bottom regions of the patch.

**Figure 20:**
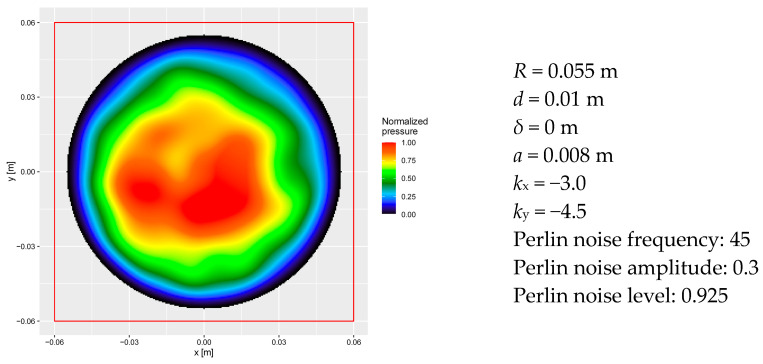
Normalized pressure map generated by the numerical model for the specified input parameters and random seed 128.

The parameter set used for the example presented below was selected from among the best-fitting configurations identified in the Monte Carlo simulations (seed = 128). The resulting pressure distribution is visualized in Figure 20, accompanied by the key model parameters defining the shape and spatial variability of the pressure field.

To evaluate the consistency between the modeled and empirical results, the mean normalized pressure and the surface area share within each predefined pressure interval were computed for the full circular dab and all previously described square sub-sections. Detailed numerical values are presented in Table 7, Table 8 and Table 9.

The surface area distribution by pressure interval for the entire adhesive dab is shown in Table 7. A comparison with the corresponding empirical distributions for Sample 2 and Sample 6 reveals three key points of agreement: (1) low RMSE and MAE values, (2) close alignment of the mean normalized pressure, and (3) similar surface area shares within the [0.975, 1] and [0.9, 1] pressure intervals.

The numerical model, configured with a mortar dab radius of *R* = 0.055 m and thickness *d* = 0.01 m, produced results that are in good agreement with the empirical pressure distributions obtained from sensor mat measurements. Comparison with Sample 2 yielded RMSE = 1.5113 and MAE = 1.0943, while for Sample 6 the corresponding values were RMSE = 1.5063 and MAE = 1.3483. These values indicate that, on average, the modeled pressure distribution deviates by approximately 1.5 percentage points per pressure interval from the empirical data. This level of accuracy is comparable to that observed for the best-fitting configurations identified through Monte Carlo simulations (see Figure 19 and related discussion).

The mean normalized pressure from the numerical model (0.5169) was compared to the grand mean value of 0.5189 obtained across both experimental samples and both types of pressure maps. This grand mean includes the average of 0.4920 from interpolated maps and 0.5458 from sensor grid maps.

The modeled results also agree well with empirical data when considering selected pressure intervals. In the [0.975, 1] range, the model yielded 5.47% of the contact area, compared to the empirical average of 4.91% (4.42% for interpolated maps and 5.41% for grid-based maps). In the broader interval [0.9, 1], the modeled area share was 12.32%, while the empirical mean was 12.35% (10.75% interpolated, 13.96% grid).

Additionally, Table 7 illustrates the effect of varying certain model parameters. Increasing the radius of the mortar dab to *R* = 0.06 m and introducing a peripheral zero-pressure ring (*δ* > 0) led to a 66.18% decrease in the mean normalized pressure. Changing the thickness of the adhesive layer from *d* = 0.01 m to *d* = 0.02 m also reduced the mean pressure, with drops of 30.80% for the *R* = 0.055 m case and 31.31% for *R* = 0.06 m. The mean normalized pressure in a 0.05 m × 0.05 m section of adhesive mortar dab, depending on its position, is presented in Table 8.

The values obtained for the adhesive mortar dab with a radius of *R* = 0.055 m and thickness *d* = 0.01 m were compared with the corresponding values derived from the empirical pressure maps for Sample 2 and Sample 6, as presented in Table 6. According to this table, the grand mean of the central section is 0.8597, while the grand mean of the side sections is 0.6700. For the considered numerical model, these values are 0.8707 and 0.6791, respectively. The corresponding errors of 1.28% and 1.36% are considered small.

Next, Table 9 presents the percentage reference of mean normalized pressure in the central section compared to other areas and the entire adhesive mortar dab.

Assuming the mean normalized pressure in the central section at a thickness of *d* = 0.01 m as the reference (100%), the mean value over the entire adhesive mortar dab is 59.37% for *R* = 0.055 m and 40.04% for *R* = 0.060 m. The first of these values may be compared with the grand mean of 60.46% obtained from the analysis of pressure maps for Sample 2 and Sample 6 (see Section 3.5.1). The difference between the numerical result (59.37%) and the experimental average (60.46%) is only 1.09 percentage points, which translates into a relative error of 1.8%. This close agreement supports the reliability of the numerical model in capturing the mean pressure distribution, even for geometrically simplified (circular) dabs.

When comparing the average pressure in the central sections of adhesive dabs with different thicknesses, the numerical model yields a value for *d* = 0.02 m that is approximately 30% of the corresponding value for *d* = 0.01 m. This result is consistent with the average drop observed in extended experimental pull-off tests on concrete substrates (approx. 25–26%; to be presented in a separate publication), although the individual samples discussed earlier in this paper (see Table 3) exhibited a more pronounced decrease (from 9 to 18%). Achieving such a reduction in the numerical model is feasible by scaling the Perlin noise level as a function of mortar thickness or, if necessary, substrate type—without altering its baseline range, which was previously established through Monte Carlo simulations for *d* = 0.01 m. However, it should be emphasized that meaningful comparisons between numerical and experimental results require that the fracture patterns observed in tests correspond to a predominantly adhesive failure mode, as cohesive or mixed failures may lead to significantly different interpretations.

In turn, when considering the adhesive mortar dab with a radius of *R* = 0.06 m from the discussed numerical example and the pull-off test results presented in Table 3 (see Section 3.4), it can be observed that while the numerical model realistically reproduces the contact pressure distribution in cases without a peripheral zero-pressure ring, the introduction of such a ring (not detectable by the sensor mat) alters the pressure pattern. In particular, the modeled side-to-center pressure ratio of around 70% is higher than the relative adhesion levels observed in the lateral zones during pull-off testing. The average bond strength in these side regions amounts to 51.5% to 52.8% (max. value 67.2%) for 10 mm thick dabs, and drops to 21.5% to 32.9% for 20 mm thick dabs when zero values are included in the mean, or 32.2% to 39.4% (max. value 50%) when those zero values are excluded. These values are expressed as percentages relative to the corresponding central region strength. Among the contributing factors to this divergence are the following:

- The effective bonded area at the edge may be smaller than assumed (the 20% *R* peripheral zero-pressure ring may underestimate the true edge effect);
- A scaling relationship between contact pressure and bond strength may be required, particularly in low-pressure edge zones, as adhesion strength may not scale proportionally with pressure in these regions.

However, it should be noted that a single numerical simulation allows for the precise extraction of average pressures at any location within the adhesive dab. In contrast, experimental measurements of bond strength in the center and side regions must typically be performed on separate physical samples. This introduces unavoidable variability stemming from sample preparation, local material differences, or test-specific boundary conditions.

Additional experimental studies targeting localized adhesion strength are needed to define correction factors or establish transfer functions between contact pressure and bond performance.

## 4. Conclusions

In real-world ETICS applications, the method of adhesive bonding between thermal insulation and the wall surface varies significantly. In situ investigations revealed that the application method of the adhesive mortar on insulation boards, the overall contact surface, and the thickness of the adhesive layer often do not comply with the manufacturers’ recommendations. The conducted tests demonstrated that the adhesive layer thickness is generally significantly greater than the recommended value of up to 10 mm. In many cases, the perimeter strip is not implemented, and only spot-applied adhesive dabs are used. It was also shown that a peripheral zone exists—both in the adhesive dabs and in the perimeter ribbon—that has no contact with the substrate or insulation, or where the contact does not result in meaningful adhesion. In adhesive dabs with a diameter of approximately 125 mm and thicknesses of 10 mm and 20 mm, this zone was estimated to extend 7–15 mm inward from the edge.

The pressure distribution tests using a sensor mat (based on the original PMAST method) revealed zones of differentiated contact pressure generated during the “bonding” of samples, both in the adhesive dabs and the perimeter ribbon. The highest contact pressures between the adhesive mortar and the substitute substrate (sensor mat surface) were observed in the central regions of both the adhesive dabs of near-optimal diameter and the perimeter ribbon. The analysis of the pressure maps at the mortar-substrate interface showed that the average pressure values corresponded to approximately half of the maximum values, while the highest pressures were concentrated within only about one-tenth of the total contact area.

The magnitude, dynamics, and distribution of the pressure applied during the bonding of insulation to the substrate influence the adhesion of the mortar to both the substrate and the insulation. The lower the adhesion in the central part of the adhesive dab, the weaker the adhesion tends to be in the surrounding zones. This observation aligns with the pressure distribution patterns revealed in the PMAST method and the bonding strength differences identified in CAST and DAST pull-off tests.

The most commonly used thermal insulation materials in ETICS, such as expanded polystyrene boards, mineral wool, or polyurethane foam, at the thicknesses currently applied, exhibit sufficient stiffness to ensure a similar distribution of pressure on the adhesive mortar during bonding to the wall. In every case of applying adhesive mortar to insulation boards, there is a phase that provides the initial tack of the adhesive mortar even before the board is pressed against the wall. Therefore, the type of thermal insulation should not have a significant influence on the adhesion of the mortar to the substrate.

The tests conducted using the original GCAT method demonstrated that when the adhesive layer exceeds 15 mm in thickness, it becomes impossible to achieve the effective bonding surface area between the insulation and the substrate required by Polish guidelines. It can also be inferred that, for thicknesses above 15 mm, the perimeter adhesive ribbon exhibits significantly lower adhesion to the substrate compared to the adhesive dabs. At a maximum tested thickness of 30 mm, the perimeter ribbon exhibited virtually no contact with the substrate, and only the dabs provided limited bonding, resulting in an overall contact area far below the minimum requirements.

These findings have practical implications, particularly for the economic planning of ETICS systems, as they highlight the critical relationship between adhesive layer thickness and material efficiency. An adhesive layer thickness of 30 mm, with an adhesive consumption of 12 kg/m^2^, should be regarded as entirely non-functional in ETICS applications. Further analysis indicates that effective adhesion cannot be achieved using the ribbon-and-dab method with cement-based mortars at layer thicknesses above 20 mm if adhesive consumption is to remain reasonable. In practice, adhesive layers are often applied at around 15 mm, but the amount of adhesive rarely reaches 12 kg/m^2^. As the GCAT tests have shown, this typically results in bonding areas that fall short of the 40% effective contact threshold considered the standard in Polish guidelines [25,27].

The conclusions drawn from adhesion tests of two mortars to concrete substrates using the original CAST and DAST methods indicate that the contact pressure applied during thermal insulation bonding has a significant impact on the adhesion of the tested mortars to the substrate. As the thickness of the adhesive layer increases, the contact pressure between the mortar and both the substrate and the insulation decreases, which in turn leads to a reduction in adhesion—particularly to the substrate.

The obtained results and conclusions may serve as a basis for continuing the initiated research on thermal insulation bonding, which may also provide inspiration for defining additional testing procedures that broaden the scope of mortar performance assessment

The CAST method investigations have been extended to a much larger group of different adhesive mortars for bonding polystyrene and various construction substrates; the results and their interpretation will be presented in a separate publication.

## Figures and Tables

**Table 1:** Summary of effective bonding surface areas between the adhesive mortar and the thermal insulation.

Insulation Board (Figure 9)	Adhesive Layer Thickness [mm]	Adhesive Perimeter Ribbons and Dabs [%]	Adhesive Perimeter Ribbons [%]	Adhesive Dabs [%]
a	20	29.9	25.4	4.5
b	10	48.7	39.3	9.4
c	18	24.2	0.0	24.2
d	15	33.5	19.3	14.2
e	13	22.4	0.0	22.4
f	21	21.9	0.0	21.9
g	11	30.1	22.6	7.5
h	9	33.5	19.3	14.2
i	7	49.3	39.9	9.5

**Table 2:** Percentage contact area between the adhesive mortar and the substitute substrate (glass plate) for various adhesive layer thicknesses. A total of 6 kg of fresh adhesive mortar was used per EPS insulation board of 100 cm × 50 cm, corresponding to 12 kg/m^2^.

Adhesive Layer Thickness [mm]	Adhesive Perimeter Ribbons and Dabs [%]	Adhesive Perimeter Ribbons [%]	Adhesive Dabs [%]
30	7.7	2.3	5.3
20	24.1	14.6	9.5
15	38.4	24.0	14.4
10	63.9	37.2	26.7

**Table 3:** Pull-off test results of samples detached from the substrate.

Adhesive Mortar 1									
Sample No.	1	2	3	1		2		3	
Sample Location	Center	Center	Center	L	R	L	R	L	R
Dab thickness 10 mm, pull-off [MPa]	1.80	1.70	1.60	0.55	0.81	1.05	1.07	0.90	0.84
Dab thickness 20 mm, pull-off [MPa]	0.16	0.21	0.18	0	0.06	0.04	0	0.05	0.08
Adhesive mortar 2									
Sample No.	1	2	3	1		2		3	
Sample location	center	center	center	L	R	L	R	L	R
Dab thickness 10 mm, pull-off [MPa]	1.41	1.24	1.31	0.87	0.58	0.58	0.56	0.72	0.88
Dab thickness 20 mm, pull-off [MPa]	0.24	0.20	0.23	0.09	0.07	0.10	0.10	0	0.07

**Table 4:** Surface area distribution by pressure range for selected samples.

Normalized Pressure Interval	Sample No. 2		Sample No. 6	
	Pressure Map with 5 × 5 mm Grid	Interpolated Pressure Map	Pressure Map with 5 × 5 mm Grid	Interpolated Pressure Map
	% of Surface Area			
[0.001, 0.1)	8.52	16.10	6.82	14.42
[0.1, 0.2)	5.57	8.55	10.23	10.22
[0.2, 0.3)	13.11	9.61	10.80	8.95
[0.3, 0.4)	8.52	7.86	7.39	7.85
[0.4, 0.5)	7.87	7.13	7.95	7.79
[0.5, 0.6)	7.54	7.72	8.81	8.60
[0.6, 0.7)	10.16	9.39	11.65	10.86
[0.7, 0.8)	12.46	11.91	13.35	12.90
[0.8, 0.9)	10.82	9.53	10.51	9.11
[0.9, 0.95)	5.90	4.21	5.40	4.50
[0.95, 0.975)	2.95	2.29	2.84	1.66
[0.975, 1]	6.56	5.69	4.26	3.14
[0.9, 1]	15.41	12.19	12.50	9.30

**Table 5:** Summary of mean pressure values and contact zone radius for selected samples.

Quantity	Sample No. 2		Sample No. 6	
	Pressure Map with 5 × 5 mm Grid	Interpolated Pressure Map	Pressure Map with 5 × 5 mm Grid	Interpolated Pressure Map
Mean radius of the contact pressure zone [m]	0.0493	0.0520	0.0529	0.0558
Mean normalized pressure [-]	0.5510	0.4957	0.5405	0.4882

**Table 6:** Mean normalized pressure in 0.05 m × 0.05 m sections of adhesive mortar dabs for two samples.

Section Position (See Figure 5)	Sample No. 2		Sample No. 6	
	Pressure Map with 5 × 5 mm Grid	Interpolated Pressure Map	Pressure Map with 5 × 5 mm Grid	Interpolated Pressure Map
Left side	0.6925	0.6640	0.7474	0.7348
Center	0.8598	0.8971	0.8242	0.8576
Right side	0.6645	0.6409	0.6395	0.6313
Top side	0.6775	0.6558	0.7506	0.7431
Bottom side	0.6809	0.6411	0.5985	0.5574
Mean of side sections	0.6789	0.6504	0.6840	0.6666

**Table 7:** Surface area distribution by pressure range for numerical sample—entire dab.

Normalized Pressure Interval	*R* = 0.055 m		*R* = 0.060 m	
	*δ* = 0		*δ* = 0.2 *R*	
	*d* = 0.01 m	*d* = 0.02 m	*d* = 0.01 m	*d* = 0.02 m
	% of Surface Area			
[0, 0.001)	0.94	1.68	36.55	36.95
[0.001, 0.1)	12.09	28.01	7.32	16.19
[0.1, 0.2)	7.87	32.13	4.80	19.15
[0.2, 0.3)	8.05	32.77	4.83	23.69
[0.3, 0.4)	8.61	5.40	5.21	4.02
[0.4, 0.5)	9.04	-	5.62	-
[0.5, 0.6)	9.56	-	5.82	-
[0.6, 0.7)	9.42	-	5.74	-
[0.7, 0.8)	11.27	-	7.87	-
[0.8, 0.9)	10.83	-	7.41	-
[0.9, 0.95)	4.39	-	3.35	-
[0.95, 0.975)	2.46	-	1.87	-
[0.975, 1]	5.47	-	3.59	-
[0.9, 1]	12.32		8.81	
Mean normalized pressure [-]	0.5169	0.1592	0.3421	0.1071

**Table 8:** Mean normalized pressure in a 0.05 × 0.05 m section of adhesive mortar dab.

Section Position (See Figure 5)	*R* = 0.055 m		*R* = 0.060 m	
	*δ* = 0		*δ* = 0.2 *R*	
	*d* = 0.01 m	*d* = 0.02 m	*d* = 0.01 m	*d* = 0.02 m
	Mean Normalized Pressure [-]			
Left side	0.7182	0.2163	0.6215	0.1900
Center	0.8707	0.2555	0.8544	0.2540
Right side	0.6581	0.2036	0.5799	0.1822
Top side	0.6123	0.1823	0.5409	0.1634
Bottom side	0.7279	0.2185	0.6308	0.1924
Mean of side sections	0.6791	0.2052	0.5933	0.1820

**Table 9:** Percentage reference of mean normalized pressure in the 0.05 m × 0.05 m central section to other sections and the entire adhesive mortar dab.

Section Position (See Figure 5)	*R* = 0.055 m		*R* = 0.060 m	
	*δ* = 0		*δ* = 0.2 *R*	
	*d* = 0.01 m	*d* = 0.02 m	*d* = 0.01 m	*d* = 0.02 m
	Mean Normalized Pressure [%]			
Left side	82.49	24.84	72.74	22.24
Center	100.00	29.34	100,00	29.73
Right side	75.58	23.38	67.87	21.32
Top side	70.32	20.94	63.31	19.12
Bottom side	83.60	25.09	73.83	22.52
Mean of side sections	78.00	23.56	69.44	21.30
Entire dab	59.37	18.28	40.04	12.54

## Data Availability

The original contributions presented in this study are included in the article and partly in the Supplementary Materials. Further inquiries can be directed to the corresponding author.

## Supplementary Materials

The following supporting information can be downloaded at: https://www.mdpi.com/article/10.3390/ma18174043/s1, Code S1: R script for the analysis of pressure-sensing mat samples (PMAST); Code S2: R script for pressure decay function fitting and optimization; Code S3: R script for Monte Carlo simulations and statistical error metrics; Code S4: R script for numerical modeling of adhesive dab pressure distribution; Dataset S1: Normalized pressure data from PMAST—Sample 2 (5 × 5 mm grid); Dataset S2: Normalized pressure data from PMAST—Sample 6 (5 × 5 mm grid); Dataset S3: Radial pressure decay profiles—full dataset (4725 points); Dataset S4: Radial pressure decay profiles—reduced dataset (582 points).

## Abbreviations

The following abbreviations are used in this manuscript:

EU	European Union
EAD	European Assessment Document
ETICS	External Thermal Insulation Composite Systems
EPS	Expanded Polystyrene
MW	Mineral Wool
TR	Tensile Strength Perpendicular to Face (EN 1607)
HBW	*Hellbezugswert* (Light Reflectance Value)
CAST	Central Adhesive Spot Test (original method)
DAST	Dual-Adhesive Spot Test (original method)
PMAST	Pressure Mapping Adhesive Spot Test (original method)
GCAT	Glass Contact Area Test (original method)
GenAI	Generative Artificial Intelligence
IDE	Integrated Development Environment
R^2^	Coefficient of Determination
RMSE	Root Mean Square Error
MAE	Mean Absolute Error
MAD	Mean Absolute Deviation
